# Ontario Veterinary College; Commencement

**Published:** 1891-05

**Authors:** 


*Ontario Veterinary College; Commencement.*—The Ontario Veterinary College closing exercises were held on Saturday morning, March 28th, in the spacious lecture hall of the fine new building on Temperance street. The Students filled the body of the hall, and the number of visitors in attendance was very large. Prof. Andrew Smith, principal of the college, presided, and beside him on the platform were: Hon. Edward Blake, Hon. A. S. Hardy, Lieut.-Col. F. C. Denison, M.P., Hon. J. B. Robinson, Mayor Clarke, Joseph Tait, M.P.P., Col. Otter, Capt. Manley, Rev. G. M. Miligan, Dr. Daniel Clarke, Sheriff Mowat, Dr. Ferguson, ex-M P, ex-Ald. Frankland, Harry Piper, Archibald Blue, Capt. McMaster, Ald. Saunders, Ald. Score, Dr. O'Reilly, Dr. Thorburn and Henry Wade.

The list of graduates was read by Dr. Duncan, and Hon. Edward Blake, in presenting the first prizes expressed himself as being very much interested in the success of the college. Much of the wealth of Ontario lies in stock, Mr. Blue put the value of the horses and cattle down at $100,000,000. He impressed on the students the great responsibility laid on their shoulders of looking after the welfare of so much wealth.

Addresses were also given by Hon. A. S. Hardy, Hon. J. B. Robinson, Lieut.-Col. Denison, M.P., Joseph Tait, M.P.P., and several others of the visitors. Many of the students at the college are from the United States, and this fact was made the subject of happy comment.

After the presentation of prizes Mr. A. C. Keelor, of Harleysville, Pa., chairman of the graduating class, in an eloquent address, presented Prof. Smith with an exceedingly handsome picture of the graduating class of 1891. Prof. Smith, in reply, thanked the students most heartily for this token of their appreciation of the curriculum and teachings of the college. He also spoke with feelings of regret at parting with a class that certainly has not been excelled in any previous year.

The board of examiners consisted of Mr. Cowan, V.S., Galt; Mr. C. Elliott, V.S., St. Catharine's; Mr. A, O. F. Coleman, Ottawa; J. D. O'Neil,

V.S., London; W. Shaw, V.S., Dayton; J. H. Wilson, V.S., London; T. H. Lloyd, V.S., Newmarket; C. H. Sweetapple, V.S., Toronto; James Thorburn, M.D., Toronto, and were assisted by C. W. Sallade, V.S., Pottsville, Pa.

The following received the degree of V. S.: John E. Alexander, Mascouche Rapids, P. Q; M. T. Anewalt, Pleasant Hills, Ohio; Edward Appleyard, Grand Valley, Ont.; David T. Augustine, Calton, Ont.

Frank M. Barnes, St. Thomas, Ont.; J. W. Barr, Milverton, Ont.; H. M. Batchelder, Warrensburg, Ill.; G. H. Bellaire, Pembroke, Ont.; James T. Boothby, Altona, Ont.; Hamilton Bowen, North Fairfield, Ohio; Percival T. Bowlby, Port Dover, Ont.; C. W. Brossman, Lower Heidelberg, Pa.; Alexander H. Brown, Pipestone, Minn.; Bruce E. Brown, St. Catharines, Ont.; William A. Brown, Pipestone, Minn.; Robert M. Bryan, Lexington, Ky.; William M. Burdick, Grovesend, Ont.; S. G. Burkholder, Denver, Pa.; Samuel Burkholder, Virden, Man.; Jame C. Bromeson, Mansfield, Ohio.

Anderson Crawforth, Brampton, Ont.; E. H. Callander, Kirkton, Ont.; Lewis W. Carl, Campchase, Ohio; William E. Carnes, Greenwood, Ind.; William R. Claussen, Waupaca, Wis.; Walter C. Clevenger, Union City, Ind.; E. J. Cobleigh, Parkhill, Ont.; S. J. Collins, Green River, Ont.; Vilroy M. Connelly, Owatonna, Minn.; Wilson S. Corliss, Carthage, N. Y.; Eli M. Crawford, Brampton, Ont.; John P. Creamer, Regina, N. W. T.; Wilton F. Crewe, Hillsboro, North Dakota; W.J. Cunnington, Parkhill, Ont.; Louis M. Cole, Toledo, Ohio.

James Drury, Toronto, Ont.; Lewis Dunn, Jr., Erie, Pa.; Joseph H. Dietz, Owatonna, Minn.; Charles E. Edmonds, Fingal, Ont.; James Cicero Everest, Arkona, Ont.; Alex. Findlay, Toronto, Ont.; George M. Fitzgerald, Chiselhurst, Ont.; J. E. Foster, Mount Eaton, Ohio; Albert A. Frank, Great Valley, N. Y.; Byron M. Freed, Sharon, Pa.

John A. Genung, Slaterville Springs, N. Y.; James Mitchell Gibb, Kingston, Jamaica, W. I.; Duncan R. Gillies, Moffat, Ont.; Whitfield Gray, Newton, N. J.; W. C. Galbraith, Brampton, Ont.; Charles H. Hackett, Linwood, Ont.; John M. Hagerman, Lynedoch, Ont.; D. C. Hanawalt, Frankfort, Ohio; J. A. Hanisch, Lake City, Minn.; J. E. Hodgins, Mooresville, Ont.; E. Burwell Holmes, London, England; J. H. Honan, Delphi, Ind.; Arthur George Hopkins, London, England; Richard Harrison, Bad Axe, Mich.; John A. Jobson, Franklin, Pa.; Herbert J. Johnston, Jaspar, Ont.; Joseph B. Johnston, Gallipolis, Ohio.; Elisha K. Kane, Warren, Ill.; William Kaul, St. Mary's, Pa.; Allen Z. Keelor, Harleysville, Penn.; James S. Kelley, Irving, Ill.; Robert L. Kelley, Irving, Ill.; Peter J. Kershner, Bernville, Pa.; Joseph E. King, Palmyra, Ill.; Joseph Brady Kinter, Marion Centre, Pa.; J. W. Klotz, Arcadia, Ind.; Foster J. Kyle, Cedarville, Ohio.

A. J. Lamberson, Whitehall, Wis.; D. C. Langford, Mason City, Iowa; Henry C. Lyon, Grandon, North Dakota; David Lewis, Omemee, Ont.

Clarence McLennan, Greenwood, Ind.; Uri Burtton McCurdy, Hutchison, Kansas; R.A. McLoughry, Wolseley, N.W.T.; W. H. McLaren, Fargo, North Dakota.

Robert McDonald, Emerson, Man.; Alex. Machan, Mitchell, Ont.; H. L. Marsack, Tunbridge Wells, Eng.; George W. Moore, Burgessville, Ont.; Andrew Smith Morrison, Bristol, P. Q.; Thomas Morrisey, DeWitt, Iowa;

John Joseph Mountford, Blenheim, Ont.; W. H. Moyer, Pottsville, Pa.; H. M. Manley, Arkansas City, Kan.

George C. Neale, Parkhill, Ont.; W. G. Neilson, Battleford, N. W. T.; W. A. Nixon, Brampton, Ont,; Hubert A. Ovens, Maple Lodge, Ont.

D. Augustus Platt, Lexington, Ky.; Charles F. Palmer, Wooster, Ohio; A. L. Parker, Providence, R. I.; J. G. Parslow, Clarinda, Iowa; James A. Pendergast, Phœnix, N. Y.: Edmund C. Porter, Waterford, Pa.; Albert R. Potteiger, Bernville, Pa.; J. O. F. Price, Sibley, Iowa; William H. Pullen, Lebanon, Ohio.

Jackson Rhodes, Uxbrldge, Ont.; Theodore S. Rich, Avon, N. Y.; George Robb, London, Ont.; Ralph B. Robinson, Brooklin, Ont.; John W. Rollings, Lancaster, S. C.; Nathaniel Robinson, Dresden, Ont.; J. L. Ronan, Auburn, N. Y.; Alexander Sanson, Petrolea, Ont.; W. H. Shadwell, Burgess Hill, Sussex, Eng.; Charles Shain, London, Ont.; Victor W. Shirley, Watford, Ont.; Septimus Sisson, Manhattan, Kas.; Alexander Esdale Smith, New Perth, P.E.I. ; Richard H. Smith, St. Mary's, Ont.; William Spence Stinson, Orangeville, Ont.; Mark A. Storey, Princeton, Ill. John B. Sowers, Greencastle, Pa.

William R. Taylor, Winnipeg, Man.; Thomas Thacker, Portage du Fort, P.Q.; Sherman L. Peeple, Napoleon, Ohio; Joseph A. Thompson, Elginfield, Ont.; Samuel Thompson, Roseneath, Ont.; S. F. Tolmie, Victoria, B.C.; W. George Turner, Point Edward, Ont.; Joshua P. Thomson, Upper Grove, Ont.; Frederick Tilt, Brampton, Ont.

Delo Vanderslice, Salem, Ohio; George B. Vliet, Hackettstown, N. J.; Hugh F. Vulliamy, Ipswich, England.

Ernest J. Walsh, Oakville, Ont.; Ulysses E. Ward, Overton, Neb.; W. J. Wadsworth, Hurray, Ont.; A. H. Wilson, St. Hary's, Ont.; Joseph E. Williams, Fingal, Ont.

The following prizes were awarded:

Pathology.—First prize, S. Sisson, silver medal; second prize, A. G. Hopkins; third prize, A. Z. Keller.

Materia Medica.—First prize, W. S. Corliss; second prize, William R. Claussen; third prize, S. G. Burkholder.

Chemistry.—First prize, J. H. Gibb; second prize, A. G. Hopkins; third prize, Harry Harsack.

Morbid Anatomy.—First prize, S. Sisson; second prize, W. J. Wadsworth; third prize, A. E. Hopkins and E. B. Holmes, equal.

Anatomy.—First prize, silver medal, S. Sisson; second prize, S. G. Burkholder; third prize, E. Appleyard.

Dissected Specimens.—Gold medal, given by Toronto Industrial Exhibition Association, awarded to Eli H. Crawford; second prize, E. J. Walsh; third prize, W. A. Nixon; fourth prize, E. C. Porter. Special prizes—J. Burneson, D. C. Gillies, R. E. Stevenson, W. J. Wadsworth.

Entozoa.—Prize, A. G. Hopkins.

Physiology.—First prize, silver medal, S. Sisson; second prize, R. H. Smith; third prize, H. F. Vulliany and W. J. Wadsworth, equal.

Best General Examination.—Jas. S. Kelly, Oregon. U.S., gold medal, presented by the Ontario Veterinary Association.

Bacteriology.—First prize, W. J. Wadsworth; second prize, S. Sisson.

Prizes for essay on Bacteriology.—First prize, Harry Harsack; second prize, E. Burwell Holmes; third prize, A. G. Hopkins.

Special prize presented by Mr. W Christie, Trustee of Toronto University, to student taking the greatest number of prizes--Complete set of Darwin's works—S. Sisson.

Special prize books presented by John Hoskin, Q.C., Trustee of Toronto University.

Physiology.—First prize, S. Sisson ; second prize, R H. Smith ; third prize, H. F Vullainy and W. J. Wadsworth, equal.

